# Behavioral Intention and Its Predictors toward COVID-19 Booster Vaccination among Chinese Parents: Applying Two Behavioral Theories

**DOI:** 10.3390/ijerph19127520

**Published:** 2022-06-20

**Authors:** Meng Zhou, Li Liu, Shu-Yan Gu, Xue-Qing Peng, Chi Zhang, Qi-Feng Wu, Xin-Peng Xu, Hua You

**Affiliations:** 1 School of Nursing, Nanjing Medical University, Nanjing 211166, China; zm0119ningmeng@163.com (M.Z.); m17839943068@163.com (C.Z.); 2 Section of School Health, Nanjing Municipal Center for Disease Control and Prevention, Nanjing 210003, China; liuli202288@126.com; 3 Center for Health Policy and Management Studies, School of Government, Nanjing University, Nanjing 211166, China; gushuyan@nju.edu.cn; 4 School of Public Health, Nanjing Medical University, Nanjing 211166, China; pengxueqing1230@163.com (X.-Q.P.); wqf@njmu.edu.cn (Q.-F.W.); xuxinpeng@njmu.edu.cn (X.-P.X.); 5 Institute of Healthy Jiangsu Development, Nanjing Medical University, Nanjing 211166, China

**Keywords:** vaccination intention, COVID-19 booster vaccination, protection motivation theory, theory of planned behavior, children

## Abstract

The booster vaccination of COVID-19 is being implemented in most parts of the world. This study used behavioral psychology to investigate the predictors of parents’ intentions regarding the COVID-19 booster vaccination for their children. This is a cross-sectional study with a self-designed questionnaire based on two behavioral theories—protective motivation theory (PMT) and theory of planned behavior (TPB). A stratified multi-stage sampling procedure was conducted in Nanjing, China, and multivariable regression analyses were applied to examine the parents’ intentions. The intention rate was 87.3%. The response efficacy (ORa = 2.238, 95% CI: 1.360–3.682) and response cost (ORa = 0.484, 95% CI: 0.319–0.732) in the PMT, were significant psychological predictors of parents’ intentions, and so were the attitude (ORa = 2.619, 95% CI: 1.480–4.636) and behavioral control (ORa = 3.743, 95% CI: 2.165–6.471) in the TPB. The findings of crucial independent predictors in the PMT and TPB constructs inform the evidence-based formulation and implementation of strategies for booster vaccination in children.

## 1. Introduction

The current coronavirus disease 2019 (COVID-19) is still spreading worldwide, in a pandemic which has lasted more than two years [1]. It is universally susceptible to all age groups and the infection incidence among children is on the rise, leading to frequent cases of severe illness or death among children [2,3]. It is estimated that about 1.7% of clinical COVID-19 infections were in under 18s in the United States, with a global infection rate of 2.0% to 4.8% [4,5,6]. In addition, educational facilities (such as kindergartens and primary schools), with a high density of people, are more prone to clusters of outbreaks [7].

Achieving herd immunity through vaccination is a cornerstone strategy in limiting the spread of epidemics [8]. It was reported by World Health Organization that more than 10.4 billion COVID-19 vaccine doses had been used worldwide until 20 February 2022 [9]. COVID-19 vaccination for children is an indispensable step in building herd immunity and constructing an immunological barrier [10]. Since the safety, tolerability, and immunogenicity of inactivated vaccines in children have been verified [11,12,13], the COVID-19 vaccine for emergency usage in 3- to 17-year-olds was formally approved in China in June 2021. Following that, basic COVID-19 vaccination for children aged 3 to 11 years old (the average age span from kindergarten to primary school) was carried out widely in China [11].

Given the continuous variation of COVID-19 and the possibility that immunity provided by the current vaccines may wane over time [14,15], the COVID-19 booster vaccination is being actively promoted by many countries and regions (such as the United States, Israel, Italy, and other developed countries) [16,17,18]. Since mid-2021 in China, priority groups (e.g., border ports, customs, medical and health staff) had received the COVID-19 booster vaccination first, followed by adults who had completed basic immunization for at least six months. Even in a few countries, such as Israel [19], children have been included in the target population of the third injection.

Nevertheless, parents’ intentions regarding their children’s COVID-19 vaccine are uneven, with a low acceptance of 59.3% and a high acceptance of 86.75% [20,21,22,23]. Hesitation towards the vaccine is pervasive. It was reported that about 33% of parents had hesitancy towards the COVID-19 vaccine for their children in the United States [20], and 35.3% in Japan [21]. Parents’ intentions play an important role in children’s booster vaccination [24]. Although there is no timetable for COVID-19 booster vaccination for children aged 3 to 11 in China, it will be necessary and forward-looking to probe the parents’ intentions to strengthen COVID-19 vaccination for children. Furthermore, identifying factors associated with intentions regarding the COVID-19 booster vaccination is urgently needed for formulating contextual-specific education and policy implementation.

The application of behavioral theories can help to understand the formation mechanism of behavior (including behavior intention) from the perspective of psychological perception [25]. The protective motivation theory (PMT) and the theory of planned behavior (TPB) are two conceptual frameworks that extensively evaluated health-promoting behaviors empirically [26,27,28], both of which have lately been utilized to predict vaccination-related intentions or behaviors [29,30]. PMT is based on the assessment of threat and response to explain the motivation of protective behavior. Threat assessment depends on: ① a person’s belief in the perceived severity of the problem (perceived severity), and ② a person’s estimation of the opportunity to experience the disease (perceived susceptibility). The coping assessment includes: ① the individual’s assessment of whether the protection behavior can effectively overcome the threat (response efficacy), ② the individual’s belief in their ability to successfully implement the protection behavior (self-efficacy), and ③ the individual’s estimate of the cost of any action (perceived response cost) [31]. TPB model assumes that attitude, subjective norm and behavior control perception will lead to the formation of behavior intention, which is considered to be the direct antecedent factor of behaviors. In general, the more favorable the attitude (i.e., positive or negative evaluation of behavior) and subjective norms (i.e., perceived social pressure), the greater the perceived control (i.e., perceived behavioral ability), and the stronger the intention of the person to perform the behavior [32]. However, the explanations or predictors identified by these behavioral theories showed controversial conclusions. For example, Huang et al. [33] discovered that coping appraisal in PMT was significantly correlated with vaccination intentions, while threat appraisal was not. However, Wang et al. [34] found the opposite results. The subjective norm in TPB was not found to be a significant predictor by Fan et al. [35], but it was significant in studies of Guidry et al. [36] and Shmueli et al. [30]. Therefore, the evidence gap for explaining parents’ behavioral intentions regarding children’s vaccination still exists and may become an obstacle to universal vaccination in practice.

This is the first study to use these two behavioral theories to analyze the parents’ intentions regarding COVID-19 booster vaccination among kindergarten and primary school children, as well as to examine the psychological perception factors independently associated with intention.

## 2. Materials and Methods

### 2.1. Data Collection

**Figure 1:**
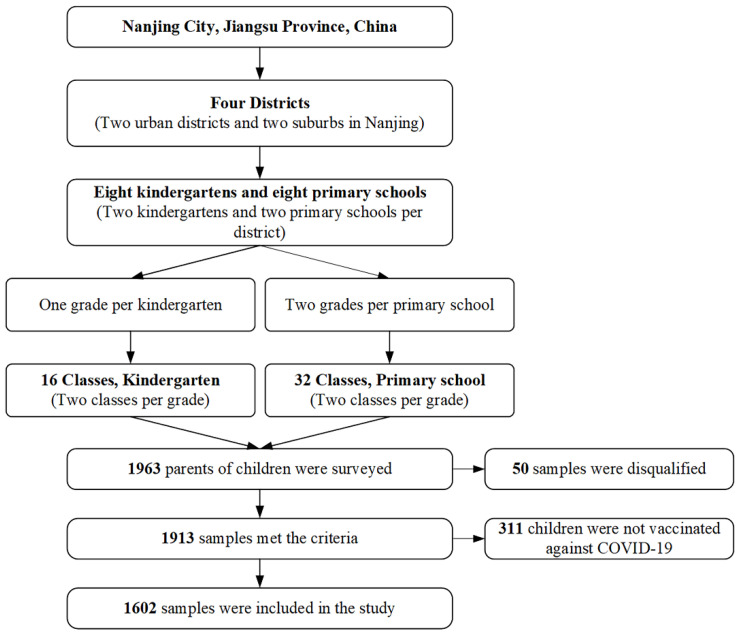
Flow chart of multistage stratified cluster sampling.

In December 2021, with multi-stage stratified cluster random sampling, an online cross-sectional survey was carried out in Nanjing, China. Nanjing is located in the economically developed coastal area of eastern China and is the capital of Jiangsu Province. In the first stage of sampling, two districts were randomly selected in the urban and suburban areas of Nanjing. In the second phase, two kindergartens and two primary schools from each district were chosen at random. In the third phase, one grade from each kindergarten was chosen, and two classes from each grade were chosen randomly; two grades were randomly selected from each primary school, and two classes were selected for each grade. (Figure 1) We used Questionnaire Star, a commonly used online survey platform in China, to post an online questionnaire. Before the survey, a question of voluntary participation was required to be answered for informed consent. Each mobile device was allowed to access the online questionnaire once to eliminate duplicate responses. For non-single-child families, participants were requested to complete the questionnaire based on the situation of the children in the questionnaire distribution class.

Finally, about 1963 participants were recruited in our study, with 50 unqualified samples being excluded and a 97.5% effective response rate. There were still 311 children who had not received a COVID-19 vaccine, so they were excluded from the study on COVID-19 booster vaccination. Finally, 1602 samples were analyzed.

### 2.2. Questionnaire Design

This study adopted a self-designed questionnaire. Our research team consisted of professionals in public health, preventive medicine, nursing, and health management. To develop the questionnaire, our research team first formulated the initial questions pool by learning the PMT and TPB framework and reviewing similar studies on vaccine intentions as well as information from the Chinese Center for Disease Control and Prevention (CCDC), the WHO, and other authoritative websites [23,37]. Through multiple discussions between study team members, we drafted the first version of the questionnaire. To ensure the content validity of the questionnaire, we modified the questionnaire based on the related literature. Then, the questionnaire was piloted among 50 parents from different schools to test whether the questions were understandable and clear. After re-review and multiple discussions in the research group, we formed the final draft version of the questionnaire. Specifically, the questionnaire consisted of three parts: (1) basic characteristics measures; (2) vaccination intention measures; and (3) PMT and TPB measures.

There were three aspects of basic characteristics measures: (1) parents’ demographic characteristics, including parents’ age, type of participants, education level, residence, marital status, and per capita monthly income; (2) children’s demographic characteristics, including the child’s age, whether they are the only child in the family, and gender; and (3) other factors that may be related to vaccination willingness including COVID-19 infection history, the child’s health status, and so on.

Parents’ intentions regarding COVID-19 booster vaccination in their children were measured by five items. Each item had a five-point Likert scale ranging from absolutely disagree to absolutely agree (from 1 to 5), with a total score of 5 to 25. A higher score indicates a stronger intention towards COVID-19 booster vaccination. We calculated Cronbach’s alpha for this scale, which was 0.998, to test the internal consistency and reliability. Bartlett sphericity test showed *p* < 0.01, Kaiser–Meyer–Olkin (KMO) was 0.871.

The measurement of psychological perception factors was designed into eight dimensions which were strictly according to the two behavioral theories. The PMT included five dimensions of psychological perceptions: severity (e.g., it would be a serious harm to child’s health if my child is infected with COVID-19); susceptibility (e.g., with increasing infection rate in children, my child is likely to be infected with COVID-19); response efficiency (e.g., after my child receives a COVID-19 booster vaccination, it can reduce the likelihood of severe outcomes); self-efficacy (e.g., I can proactively collect information about the COVID-19 booster vaccination and judge its authenticity and credibility); and response cost (e.g., the adverse effects of the COVID-19 booster vaccination may interfere with the children’s daily activities). The TPB measures included three dimensions: attitude (e.g., vaccination booster is an important means of disease prevention and herd immunity, so people should actively participate in vaccination); subjective norms (e.g., I will give my children booster vaccination due to media publicity); and behavioral control (e.g., If I want to take my child to uptake a COVID-19 booster vaccine, I could do it easily). Each dimension had five items and each item with a five-point Likert scale similar to the intention measurement. Item 4 of the attitude dimension in TPB was set reversely. Cronbach’s alpha coefficient of each dimension in the scale ranged from 0.729 to 0.966. Bartlett sphericity test showed *p* < 0.01, KMO range of the psychological perception factors is 0.791–0.899.

### 2.3. Statistical Analysis

In the data recoding process of each item, “absolutely agree” and “agree” are encoded as 1, and the rest are encoded as zero. Following that, all of the items were summed for each dimension, and a score of three or more was recognized as evidence of that perception, while a score of two or less was classified as no evidence of such perception. For parents’ vaccination intentions, the same recoding procedure was followed: a score of three or more indicates intention, but a score of two or less indicates no intention.

In the statistical analyses, binary logistic regression analysis was conducted with vaccination intention as the dependent variable. According to the hypothesis, the psychological perception factors were the key independent variables. After the univariate analyses, all psychological perception factors were included in one multivariate logistic regression model without any confounding variable. Next, one to three types of relevant covariates were successively included in three regression models to adjust the parameters of the core independent covariates. Model 1: parents’ characteristics were included as the covariates. Model 2: based on Model 1, children’s characteristics were included as covariates. Model 3: based on Model 2, other variables related to vaccination intentions were included as covariates. The above models were all conducted by the entering method, and Hosmer–Lemeshow (H–L) test was used to evaluate the degree of fit. The value of χ^2^ is small, and *p* > 0.05 indicates that the model fits well [38].

Data analysis was performed using SPSS version 25.0 for Windows (SPSS Inc., Chicago, IL, USA), with the statistical level set at *p* < 0.05 (two-sided).

## 3. Results

### 3.1. Demographic Characteristics and Other Intention-Related Factors

(Table 1) Among 1602 participants, the majority were mothers (75.9%). The median age of parents was 32 years old, and 916 had an education above college (57.2%). The median age of the children was seven years old, with 972 (60.7%) aged seven years or younger. The number of children without siblings in one family was 895 (55.9%). There were 73.4% of children who had received two doses of the COVID-19 vaccine and 26.6% had received only one dose.

### 3.2. Parents’ Psychological Perception

(Table 2) Regarding the five PMT psychological perception factors, more than half of parents had the perceptions of severity (88.3%), susceptibility (62.2%), response efficacy (82.5%), self-efficacy (86.0%), while only 25.2% responded to cost. For TPB factors, the proportion of parents with three psychological perceptions was 86.6% (attitude), 69.9% (subjective norms), and 81.1% (behavioral control).

### 3.3. Parents’ Intentions Regarding Booster Vaccination in Children

(Table 3) More than 80% of parents demonstrated the intention to actively respond to advocacy and follow up information on booster vaccination for children (such as the vaccination process and post-vaccination precautions). A total of 1398 parents reported that they were willing to let their children receive a COVID-19 booster vaccination after six months of basic immunization, of which the intention rate is 87.3%, and 12.7% of parents showed no intention.

### 3.4. Univariate and Multivariate Analysis of Intention and Psychological Perceptions

(Table 4) Univariate analysis showed that the parents’ intentions regarding COVID-19 booster vaccination in their children were related to their psychological perceptions (*p* < 0.05) including susceptibility, response efficacy, self-efficacy, attitude, subjective norms, and behavioral control. The multivariate logistic regression model showed a significant correlation between four perception factors (*p* < 0.05). Response efficacy (OR = 2.246, 95% CI: 1.391–3.627), attitude (OR = 2.415, 95% CI: 1.407–4.147), and behavioral control (OR = 3.456, 95% CI: 2.023–5.902) were positively associated with parents’ intentions. However, response cost had a negative association with the intentions among parents (OR = 0.515, 95% CI: 0.345–0.771). The result of the H–L test showed that the model fitted well (χ^2^ = 6.538, *p* = 0.479).

### 3.5. Covariates-Adjusted Multivariate Analysis of Intention and Psychological Perceptions

(Table 5) In the multivariate regression models adjusted by adding three types of covariates, the significance of the four psychological perceptions remained stable. In Model 3, the results showed that response efficacy (ORa = 2.238, 95% CI: 1.360–3.682), attitude (ORa = 2.619, 95% CI: 1.480–4.636), and behavioral control (ORa = 3.743, 95% CI: 2.165–6.471) remained positively correlated with parents’ intentions; whilst response cost (ORa = 0.484, 95% CI: 0.319–0.732) again proved to be a negative factor associated with intention. The H–L test results of the three models (Models 1, 2 and 3) showed that χ^2^ was 3.602 (*p* = 0.891), 10.033 (*p* = 0.263) and 6.910 (*p* = 0.546), respectively. These three models were all fitted well.

## 4. Discussion

The proportion of parents with the intention of getting the booster vaccination for their children in this study was more than 85%. The proportion is also similar to that of a study on parents’ intentions to vaccinate children with the basic COVID-19 vaccine (86.75%) in December 2020 [39]. Moreover, it is higher than the 75.2% willingness rate of booster vaccination in the Chinese sample reported by one study in April 2021 [40] and 76.8% willingness rate reported by another study in May 2022 [41]. In the US, 61.8% willingness rate regarding the booster vaccination was reported in July 2021 [42]. Achieving herd immunity depends on actual vaccination coverage rates. It has been estimated that, if herd immunity is to be achieved, when the vaccine effectiveness reaches 80% the global vaccination rate must reach 78.0%, and the vaccination rate in China must reach 72.9%; when the effectiveness of the COVID-19 vaccine is only 70%, the global vaccination rate must reach 89.2%, and the vaccination rate in China must rise to 83.3% [43]. However, according to the statistics of Jiangsu Provincial Health Commission in November 2021, even the vaccination rate for the first injection among the population aged 3 to 11 in Jiangsu Province is only 61% [44]. Improving vaccination rates, especially booster vaccination among specific groups such as children, will be a huge challenge in a populous country such as China.

A few similar studies have discussed this issue before, generally based on a single theory [33,34,35]. When explaining parents’ behavioral willingness to vaccinate children, in addition to personal assessment of threats and independent response, external impacts from society should also be taken into account in combination with environmental characteristics; the individual’s judgment of behavior and control over the implementation of behavior should also be included. This study combines two behavioral theories to contribute to a broader perspective in explaining the factors behind parents’ acceptance of children’s booster vaccination. It was identified that psychological perceptions based on PMT (response efficacy, response cost) and TPB (attitude, behavior control) could predict parents’ intentions.

Response efficacy, attitude, and behavioral control were positively correlated with the intention. By contrast, response cost was the negative predictor. The combination of PMT and TPB in the study for explaining and predicting health-related behaviors can complement each other and improve the degree of interpretation. The findings can provide crucial insights into potential directions for future research and guide the actions to improve COVID-19 booster vaccination coverage. The intervention strategies should consider how to improve the parents’ psychological perceptions of response efficacy, attitude, and behavioral control, as well as how to reduce the response cost.

In this study, the coping appraisal (response efficacy and response cost) in PMT theory were independent predictors, which was similar to previous studies [33,41,45,46]. Parents with high response efficacy were 2.23 times more likely than those with low response efficacy to accept COVID-19 booster vaccination for their child, indicating a positive predictive effect. This is consistent with a previous study conducted in the United States, which showed that response efficacy is the best predictor of COVID-19 vaccination acceptance [47]. Compared with general vaccines, the COVID-19 vaccine has a shorter research development cycle. Although governments all over the world hope to minimize the transmission through extensive vaccination, there may be widespread concern about the safety of short-term vaccine development [34]. It is reported that the doubts about the effectiveness and side effects are the most common reasons for parents to refuse to vaccinate their children [23,48]. Combined with the coping appraisal found in this study, it is suggested that educational institutions and disease control institutions jointly carry out intervention actions to provide parents with open and transparent vaccine information and guidance to distinguish chaotic information, so as to reduce the negative impact of vaccine safety doubts on parents’ intentions. The public health departments can also make unified planning, and increase the number of vaccination sites, so as to reduce the perceived response cost [45].

In addition, self-efficacy is also a valid role in intention formation and behavior generation [34,49]. However, it was not found to have significant impact on the parents’ intentions in this study. This finding is consistent with results from the study of Wang et al. [34] yet different from the studies of Eberhardt et al. [50] and Wu et al. [41]. Therefore, the prediction of parental self-efficacy on their intentions regarding children’s vaccination needs to be further studied.

The threat appraisal (severity and susceptibility) in PMT factors were reported to be predictive of COVID-19 vaccination intentions previously [51]. Nevertheless, these variables were not significant in our study. With the COVID-19 outbreak continuing, the public’s perception of the threat may differ from the early stages of the epidemic. As a result, public attention may have shifted from the lethality and severity of the disease to practical responses, such as vaccination evaluation. Furthermore, the Chinese government has always adhered to the dynamic zero clearing policy and has made great efforts to prevent and control new infections. Therefore, the threat appraisal of COVID-19 infection may not be an effective predictor of vaccination intentions [46]. Therefore, health promotion strategies of strengthening vaccination for children may be less effective only from the perspective of parents’ perceptions of severity and susceptibility. This finding may have enlightening value for some areas where the epidemic has been controlled at a low level.

Findings from this study revealed that attitude and behavioral control in TPB factors were both positive predictors in the COVID-19 booster vaccination intentions of parents to their children. These findings were consistent with the study of COVID-19 basic immunity by Zhang et al. [37], Guidry et al. [36], Xiao et al. [52], and Hayashi et al. [53]. Interestingly, even in different study samples, behavioral control showed consistent significance, which revealed behavioral control as a powerful predictor of COVID-19 vaccination intentions. Recently, in a booster vaccination study, attitude and subjective norms were also found to be related to intentions [54]. In order to enhance uptake of the vaccine to ensure herd immunity, and reduce the incidence of severe cases, the public health departments should raise parents’ health beliefs and social responsibility through positive publicizing of the effectiveness and social worth of the vaccine booster. Emphasizing the benefits of vaccination to individuals and the population will help to generate a positive attitude among parents toward prevention behaviors. To eliminate their hesitation, educational facilities could help parents completely understand the details of COVID-19 vaccination through offering workshops, lectures, posters, videos, and so on, so that professional content can be guided and provided by health departments.

There was no significant correlation between subjective norms in TPB factors and vaccination intentions in this study, which aligns with Fan et al. [35] and contradicts Guidry et al. [36]. In the context of widespread booster vaccination, the media publicity and family members’ attitudes (or other influential persons) may not have much impact on parents’ perceptions [35]. Thus, subjective norms may not show predictability of parents’ COVID-19 booster vaccination intentions to children in this stage.

However, this study still has several limitations: (1) this study was only conducted in a provincial capital city in eastern China. Considering the differences in epidemic prevalence and vaccination policies between different countries or regions, the results of this study should be carefully popularized. (2) This study is a cross-sectional study. Due to the lack of time factor in data collection, there is insufficient evidence to infer causality. In addition, recall bias usually exists in cross-sectional studies and should be taken into account when interpreting the results. (3) The data collection method of this study is parents’ self-reports assisted by educational institutions. Parents may tend to report higher willingness, which may also cause bias.

## 5. Conclusions

In summary, most parents have the intention to get the booster vaccination for their children in China. Regarding the PMT and TPB as the theoretical framework, this study confirmed that parents’ psychological perceptions, including response efficacy, response cost, attitude, and behavior control, are predictors of willingness to strengthen their children’s immunization against COVID-19. Despite some limitations, the findings of this study provided some implications for the implementation of related health education and intervention to promote children’s booster vaccination.

## Figures and Tables

**Table 1:** Demographic characteristics and other possible factors related to booster vaccination intention (*n* = 1602).

Variables	Number (*n*)/M(Q)	Percentage (%)
**Demographic characteristics of parents**		
Parents’ age (years) ^a^	32.0 (34.0, 35.0)	
below 34	673	42.0
34 and above	929	58.0
Type of participants		
Father or other parent	386	24.1
Mother	1216	75.9
Education		
College or below	686	42.8
College and above	916	57.2
Residence		
Urban	211	13.2
Rural	1391	86.8
Marital status		
Unmarried, divorced, widowed	65	4.1
Married	1537	95.9
Per capita monthly income (RMB) ^b^		
Less than RMB 15,000	1073	67.0
RMB 15,000 and above	529	33.0
**Demographic characteristics of children**		
Children’s age (years) ^c^	7.0 (6.0, 8.0)	
7 years and below	972	60.7
8 years and older	630	39.3
Whether is the single-child family		
No	707	44.1
Yes	895	55.9
Gender		
Boy	837	52.2
Girl	765	47.8
**Other intention related factors**		
Family member had been quarantined due to COVID-19 containment		
No	1510	94.3
Yes	92	5.7
Family member had been infected with COVID-19		
No	1599	99.8
Yes	3	0.2
Family member had been involved in COVID-19 prevention and control efforts		
No	1308	81.6
Yes	294	18.4
Parents’ COVID-19 vaccinations		
Vaccinated three doses	440	27.5
Vaccinated two doses	1079	67.4
Vaccinated one dose	29	1.8
Not vaccinated	54	3.4
This child had been vaccinated against self-funded vaccines (e.g., influenza vaccine, chickenpox vaccine, hand-foot-and-mouth disease vaccine, etc.)		
No	597	37.3
Yes	1005	62.7
Health status of child		
Good and below	215	13.4
healthy	1387	86.6
This child had respiratory or gastrointestinal issues in the last month		
No	1334	83.3
Yes	268	16.7
This child had an allergy history		
No	1355	84.6
Yes	247	15.4
This child had any contraindication to the COVID-19 vaccine		
No	1389	86.7
Yes/Unclear	213	13.3
Child’s COVID-19 vaccinations		
Vaccinated two doses	1176	73.4
Vaccinated one dose	426	26.6

^a^ Thirty-four years was a median age of parents; ^b^ income: per capita monthly income, and RMB 15,000 was the median income; ^c^ seven years was a median age of children; Abbreviations: number (*n*); median (M); interquartile spacing (Q); percentage (%).

**Table 2:** Parents’ psychological perception of PMT and TPB (*n* = 1602).

Variables	Number (*n*)	Percentage (%)
**PMT factors**		
Severity		
No	188	11.7
Yes	1414	88.3
Susceptibility		
No	605	37.8
Yes	997	62.2
Response efficacy		
No	280	17.5
Yes	1322	82.5
Self-efficacy		
No	225	14.0
Yes	1377	86.0
Response cost		
No	1199	74.8
Yes	403	25.2
**TPB factors**		
Attitude		
No	214	13.4
Yes	1388	86.6
Subjective norms		
No	483	30.1
Yes	1119	69.9
Behavioral control		
No	302	18.9
Yes	1300	81.1

**Table 3:** Parents’ intention on COVID-19 booster vaccination in children (*n* = 1602).

Variables	Number (*n*)	Percentage (%)
Having intention to get your child a booster vaccination		
Absolutely disagree	37	2.3
Disagree	34	2.1
Neutrality	229	14.3
agree	670	41.8
Absolutely agree	632	39.5
Having intention to actively respond to advocacy on booster vaccination for children		
Absolutely disagree	33	2.1
Disagree	24	1.5
Neutrality	224	14.0
agree	677	42.3
Absolutely agree	644	40.2
Having intention to actively follow up information on booster vaccination for children		
Absolutely disagree	37	2.3
Disagree	11	0.7
Neutrality	164	10.2
agree	702	43.8
Absolutely agree	688	42.9
Having intention to proactively learn the process of booster vaccination in children		
Absolutely disagree	34	2.1
Disagree	13	0.8
Neutrality	162	10.1
agree	702	43.8
Absolutely agree	691	43.1
Having intention to proactively understand the precautions for children after vaccination for booster needle		
Absolutely disagree	35	2.2
Disagree	10	0.6
Neutrality	139	8.7
agree	683	42.6
Absolutely agree	735	45.9
Intention ^a^		
Yes	1398	87.3
No	204	12.7

^a^ In the intention dimension, “absolutely agree” and “agree” are encoded as one, and the rest are encoded as zero in each item. Following that, five items were summed up, and a score of three or more indicates intention, but a score of two or less indicates no intention.

**Table 4:** Univariate and multivariate analysis of intention and psychological perceptions (*n* = 1602).

Variables	Intention *n* (%)		Univariate Analysis		Multivariate Analysis	
	No	Yes	OR (95% CI)	*p* Value	OR (95% CI)	*p* Value
Severity						
No	32 (17.0)	156 (83.0)	1	0.061	1	0.154
Yes	172 (12.2)	1242 (87.8)	1.481 (0.981, 2.237)		0.683 (0.405, 1.154)	
Susceptibility						
No	108 (17.9)	497 (82.1)	1	<0.001	1	0.858
Yes	96 (9.6)	901 (90.4)	2.039 (1.517, 2.742)		0.963 (0.642, 1.447)	
Response efficacy						
No	107 (38.2)	173 (61.8)	1	<0.001	1	0.001
Yes	97 (7.3)	1225 (92.7)	7.811 (5.686, 10.730)		2.246 (1.391, 3.627)	
Self-efficacy						
No	87 (38.7)	138 (61.3)	1	<0.001	1	0.336
Yes	117 (8.5)	1260 (91.5)	6.789 (4.889, 9.429)		1.282 (0.773, 2.125)	
Response cost						
No	149 (12.4)	1050 (87.6)	1	0.525	1	0.001
Yes	55 (13.6)	348 (86.4)	0.898 (0.644, 1.252)		0.515 (0.345, 0.771)	
Attitude						
No	102 (47.7)	112 (52.3)	1	<0.001	1	0.001
Yes	102 (7.3)	1286 (92.7)	11.482 (8.209, 16.061)		2.415 (1.407, 4.147)	
Subjective norms						
No	128 (26.5)	355 (73.5)	1	<0.001	1	0.428
Yes	76 (6.8)	1043 (93.2)	4.948 (3.635, 6.735)		1.211 (0.755, 1.943)	
Behavioral control						
No	122 (40.4)	180 (59.6)	1	<0.001	1	<0.001
Yes	82 (6.3)	1218 (93.7)	10.067 (7.306, 13.874)		3.456 (2.023, 5.902)	

Abbreviations: OR, odds ratio; CI, confidence interval.

**Table 5:** Results of multivariate logistic regression model adjusted with covariates (*n* = 1602) ^a^.

Variables	Model 1		Model 2		Model 3	
	ORa (95% CI)	*p* Value	ORa (95% CI)	*p* Value	ORa (95% CI)	*p* Value
Severity						
No	1	0.175	1	0.242	1	0.340
Yes	0.689 (0.402, 1.181)		0.722 (0.419, 1.246)		0.764 (0.440, 1.328)	
Susceptibility						
No	1	0.828	1	0.800	1	0.737
Yes	1.046 (0.694, 1.577)		1.055 (0.698, 1.593)		1.074 (0.707, 1.634)	
Response efficacy						
No	1	0.002	1	0.002	1	0.002
Yes	2.170 (1.334, 3.530)		2.142 (1.312, 3.498)		2.238 (1.360, 3.682)	
Self-efficacy						
No	1	0.320	1	0.385	1	0.387
Yes	1.297 (0.777, 2.164)		1.257 (0.750, 2.108)		1.261 (0.745, 2.135)	
Response cost						
No	1	0.001	1	0.001	1	0.001
Yes	0.501 (0.334, 0.751)		0.491 (0.326, 0.738)		0.484 (0.319, 0.732)	
Attitude						
No	1	0.001	1	0.001	1	0.001
Yes	2.474 (1.427, 4.288)		2.641 (1.516, 4.600)		2.619 (1.480, 4.636)	
Subjective norms						
No	1	0.414	1	0.513	1	0.764
Yes	1.220 (0.757, 1.966)		1.174 (0.727, 1.895)		1.077 (0.662, 1.755)	
Behavioral control						
No	1	<0.001	1	<0.001	1	<0.001
Yes	3.562 (2.073, 6.119)		3.680 (2.139, 6.333)		3.743 (2.165, 6.471)	

^a^ Only the psychological perception factors are listed in the table; Abbreviations: ORa, adjusted odds ratio; CI, confidence interval; Model 1: parents’ demographic characteristics were included as covariates; Model 2: demographic characteristics of parents and their children were included as covariates; Model 3: demographic characteristics of parents and their children, as well as other intention-related factors, were included as covariates.

## Data Availability

The dataset supporting the conclusions of this article is available from the corresponding author on reasonable request.
